# Clinical Evaluation of Three Sample-to-Answer Platforms for Detection of SARS-CoV-2

**DOI:** 10.1128/JCM.00783-20

**Published:** 2020-07-23

**Authors:** Wei Zhen, Elizabeth Smith, Ryhana Manji, Deborah Schron, Gregory J. Berry

**Affiliations:** a Infectious Disease Diagnostics, Northwell Health Laboratories, Lake Success, New York, USA; b Department of Pathology and Laboratory Medicine, The Donald and Barbara Zucker School of Medicine at Hofstra/Northwell, East Garden City, New York, USA; Boston Children's Hospital

**Keywords:** COVID-19, EUA, SARS-CoV-2, molecular diagnostics, nasopharyngeal, near-patient testing

## Abstract

Severe acute respiratory syndrome coronavirus 2 (SARS-CoV-2) has now spread across the globe. As part of the worldwide response, many molecular diagnostic platforms have been granted emergency use authorization (EUA) by the Food and Drug Administration (FDA) to identify SARS-CoV-2 positive patients. Our objective was to evaluate three sample-to-answer molecular diagnostic platforms (Cepheid Xpert Xpress SARS-CoV-2 [Xpert Xpress], Abbott ID NOW COVID-19 [ID NOW], and GenMark ePlex SARS-CoV-2 Test [ePlex]) to determine analytical sensitivity, clinical performance, and workflow for the detection of SARS-CoV-2 in nasopharyngeal swabs from 108 symptomatic patients. We found that Xpert Xpress had the lowest limit of detection (100% detection at 100 copies/ml), followed by ePlex (100% detection at 1,000 copies/ml), and ID NOW (20,000 copies/ml). Xpert Xpress also had highest positive percent agreement (PPA) compared to our reference standard (98.3%) followed by ePlex (91.4%) and ID NOW (87.7%). All three assays showed 100% negative percent agreement (NPA). In the workflow analysis, ID NOW produced the lowest time to result per specimen (∼17 min) compared to Xpert Xpress (∼46 min) and ePlex (∼1.5 h), but what ID NOW gained in rapid results, it lost in analytical and clinical performance. ePlex had the longest time to results and showed a slight improvement in PPA over ID NOW. Information about the clinical and analytical performance of these assays, as well as workflow, will be critical in making informed and timely decisions on testing platforms.

## Abstract (precis)

Severe acute respiratory syndrome coronavirus 2 (SARS-CoV-2) has now spread across the globe. As part of the worldwide response, many molecular diagnostic platforms have been granted emergency use authorization (EUA) by the Food and Drug Administration (FDA) to identify SARS-CoV-2 positive patients. Our objective was to evaluate three sample-to-answer molecular diagnostic platforms (Cepheid Xpert Xpress SARS-CoV-2 [Xpert Xpress], Abbott ID NOW COVID-19 [ID NOW], and GenMark ePlex SARS-CoV-2 Test [ePlex]) to determine analytical sensitivity, clinical performance, and workflow for the detection of SARS-CoV-2 in nasopharyngeal swabs from 108 symptomatic patients.

## INTRODUCTION

The outbreak of severe acute respiratory syndrome coronavirus 2 (SARS-CoV-2) and subsequent cases of coronavirus disease 2019 (COVID-19) (1), which began in Wuhan, China, had spread to more than 200 countries and territories by the end of December 2019. As of 15 April 2020, over two million cases have been confirmed, causing over ∼133,000 deaths according to the Centers for Disease Control and Prevention (CDC) and the database of the Center for Systems Science and Engineering (CSSE) at Johns Hopkins University (2, 3).

SARS-CoV-2 is the seventh coronavirus known to be transmitted from human to human, has high rates of transmission, and is also relatively stable in aerosols and on surfaces (4–6). Infection with SARS-CoV-2 can cause mild to severe respiratory illness, including symptoms such as fatigue, shortness of breath, cough, and fever. In addition, some individuals experience rapidly progressive and severe disease. The elderly and those with serious underlying medical conditions (e.g., cardiovascular disease, diabetes, lung disease, and immunocompromised individuals) are most at risk of developing fulminant disease (4). Currently, there are no available specific therapeutics or vaccines approved by the FDA for treatment or prevention of COVID-19 (7). In addition, the SARS-CoV-2 pandemic has coincided with influenza season in many locations. These challenges have presented a major hurdle for slowing the global spread of disease and have necessitated the need for rapid and accurate SARS-CoV-2 diagnostic testing to implement effective infection control measures.

Currently available molecular diagnostics platforms include several sample-to-answer platforms that have been issued an emergency use authorization (EUA) by the FDA to qualitatively detect SARS-CoV-2 RNA in symptomatic patients. All three sample-to-answer platforms evaluated in this study are individual cartridge-based tests that are likely to be widely utilized by hospital laboratories. In addition, both Xpert Xpress and ID NOW are also authorized for use in patient care settings outside the clinical laboratory environment and are therefore highly likely to be considered for patient testing in the outpatient environment.

In this study, our objective was to evaluate the analytical and clinical performance as well as the workflow of these three sample-to-answer platforms for SARS-CoV-2 detection in 108 nasopharyngeal (NP) swab specimens from symptomatic patients.

## MATERIALS AND METHODS

### Specimen collection and storage.

Nasopharyngeal (NP) swabs were collected from symptomatic patients. A sterile swab made from Dacron, rayon, or nylon was used for each collection. The NP swab was then placed into sterile 3-ml universal transport medium (UTM) (various manufacturers). Samples were then transported and tested as close to the time of collection as possible. The specimens were stored at 2 to 8°C for up to 72 h. Following routine patient testing, samples were aliquoted and stored at –80°C until comparator testing could occur.

### Study design.

A total of 108 nasopharyngeal samples (50 negative and 58 positive specimens) tested between March to April of 2020 were selected for this study and included samples from symptomatic patients of all genders and ages. This work was conducted as a quality improvement activity in order to complete each assay validation. The 108 specimens included 88 retrospective samples initially tested on ePlex and then immediately aliquoted and frozen at –80°C, remaining frozen until this study was performed. Retrospective samples were thawed and immediately tested using the Hologic Panther Fusion SARS-CoV-2 assay (reference standard) and the ID NOW and Xpert Xpress assays. The prospective 20 specimens were processed fresh on each platform at the time of patient testing. The specimens selected represented our true positivity rate at the time that this study was performed (50 to 60%) and also included positive specimens spanning the range of positivity levels, including those with low viral loads (characterized by high cycle threshold [*C_T_*] values obtained by the reference method).

### Cepheid Xpert Xpress SARS-CoV-2 assay.

The Xpert Xpress assay is a molecular *in vitro* diagnostic test utilizing widely used real-time reverse transcription PCR (RT-PCR) amplification technology to detect the nucleocapsid gene (N2) and the envelope gene (E) in upper respiratory specimens and is performed on the GeneXpert instrument system. All testing was performed according to the manufacturer’s instructions. Briefly, the contents of the specimen collection tube are mixed by rapidly inverting the tube five times and then 300 μl of NP specimen is transferred to the sample chamber of the assay cartridge. The lid is then closed and the cartridge is loaded onto the GeneXpert platform, which performs automated sample processing and real-time RT-PCR for viral RNA detection.

### Abbott ID NOW COVID-19 assay.

ID NOW is a rapid molecular *in vitro* diagnostic test utilizing isothermal nucleic acid amplification technology to detect the RNA-dependent RNA polymerase (RdRp) gene segment of SARS-CoV-2 and is performed on the ID NOW instrument. It consists of a sample receiver containing elution/lysis buffer, a test base, and a transfer cartridge for transfer of the eluted sample to the test base and ID NOW instrument. All testing was performed according to the manufacturer’s instructions. Briefly, a test base and a sample receiver are inserted into the ID NOW instrument. When the operator is prompted to do so via on-screen instructions, 200 μl of NP specimen is added to the sample receiver and then immediately transferred to the test base using the provided transfer cartridge, initiating target amplification.

### GenMark ePlex SARS-CoV-2 assay.

The ePlex assay is an *in vitro* diagnostic test that targets the N gene of SARS-CoV-2 and uses a combination of electrowetting and GenMark’s eSensor technology for extraction, amplification, and detection using competitive DNA hybridization and electrochemical detection. All testing was performed according to the manufacturer’s instructions. Briefly, the specimen is initially subjected to vortex mixing, after which 200 μl of NP specimen is added to the sample delivery device (SDD) provided with the ePlex SARS-CoV-2 kit and subjected to vortex mixing for 10 s. The entire volume of the SDD is dispensed into the sample loading port of the SARS-CoV-2 test cartridge, followed by pushing down the cap to seal the sample delivery port. The cartridge is bar-coded and scanned with the ePlex instrument barcode scanner and is then loaded into an available ePlex bay, which performs extraction, amplification, and detection.

### Hologic Panther Fusion SARS-CoV-2 assay (reference standard assay).

The Fusion SARS-CoV-2 assay was used as the reference standard for all three assays evaluated in this study and was performed according to the manufacturer’s instructions for use. NP specimens are lysed by transferring 500 μl of specimen into a specimen lysis tube containing 710 μl lysis buffer and loaded onto the instrument. An internal control is added to each specimen by the use of working Panther Fusion Capture Reagent-S, and hybridized nucleic acid is then separated using a magnetic field. Following wash steps, 50 μl of purified RNA is eluted. Then, 5 μl of eluted nucleic acid is transferred to a Panther Fusion reaction tube. The Fusion SARS-CoV-2 assay amplifies and detects two conserved regions of the ORF1ab gene in the same fluorescence channel, with amplification of either or both regions leading to a production of a fluorescent ROX signal. Reporting of a positive specimen requires only one of the two targets to be detected (ORF1a or ORF1b gene).

### Analytical sensitivity.

Limit-of-detection (LoD) analyses were performed using an Exact Diagnostics synthetic RNA quantified control (SARS-CoV-2 standard) containing five gene targets (E, N, ORF1ab, RdRP, and S genes of SARS-CoV-2) (stock-keeping unit [SKU] COV019; Exact Diagnostics, Fort Worth, TX). A starting concentration of 200,000 copies/ml control was used to prepare a serial dilution panel. The control material was prepared using Ambion RNA storage solution (catalog no. AM7001; Thermo Fisher Scientific) to limit the potential of degradation of the RNA transcript and aliquoted for testing to obtain replicates at 20,000, 10,000, 5,000, 2,000, 1,000, 500, 100, 50, and 5 copies/ml (with numbers of replicates ranging from 1 to 10, as shown in Table 1). The positive rate value was defined as the lowest dilution at which all replicates were positive at a 100% detection rate and was used to evaluate the analytical sensitivity of all three sample-to-answer platforms.

**TABLE 1:** Summary of limit-of-detection results

Molecular assay	Gene	No. of replicates detected at each dilution/total no. of replicates at indicated no. of copies/ml (% positive rate)^*a*^									Final LoD (no. of copies/ml)^*b*^
		20,000	10,000	5,000	2,000	1,000	500	100	50	5	
Xpert Xpress	N2	1/1 (100)	N/A	N/A	10/10 (100)	**9/9 (100)**	9/10 (90)	7/10 (70)	4/8 (50)	0/5 (0)	**100**^*c*^
	E	1/1 (100)	N/A	N/A	10/10 (100)	9/9 (100)	10/10 (100)	**10/10 (100)**	7/8 (87.5)	0/5 (0)	

ID NOW	RdRp	**5/5 (100)**	8/10 (80)	5/10 (50)	5/10 (50)	0/8 (0)	0/1 (0)	0/1 (0)	0/1 (0)	0/1 (0)	**20,000**

ePlex	N	10/10 (100)	N/A	N/A	10/10 (100)	**9/9 (100)**	7/10 (70)	1/10 (10)	1/4 (25)	0/4 (0)	**1,000**

a The limit of detection by positive rate for each gene target is highlighted in bold. N/A, not analyzed.

b The final LoD was based on each manufacturer’s results interpretation algorithm.

c Also includes presumptive positive results.

### Statistical methods.

The reference standard was established as the result obtained from the Hologic Panther Fusion SARS-CoV-2 assay. Percent positive agreement (PPA), percent negative agreement (NPA), positivity rate, Kappa, and two-sided (upper/lower) 95% confidence interval (CI) values were calculated using Microsoft Office Excel 365 MSO software (Microsoft, Redmond, WA). Cohen’s kappa values (κ) were calculated as a measure of overall agreement, with values categorized as representing almost perfect results (values of >0.90), strong results (0.80 to 0.90), moderate results (0.60 to 0.79), weak results (0.40 to 0.59), minimal results (0.21 to 0.39), or no results (0 to 0.20) (8, 9). The dose-response 95th percentile (with 95% confidence interval [CI]) model was assessed using Finney and Stevens calculations (10).

## RESULTS

### Analytical sensitivity.

LoD was determined by preparing serial dilutions ranging from 20,000 to 5 copies/ml using a known concentration of the Exact Diagnostics SARS-CoV-2 control panel and was defined as the minimum concentration with detection of 100% by positive rate. The LoD established by percent positive rate and the manufacturer’s interpretation algorithm for each assay was determined to be 20,000 copies/ml for ID NOW, 1,000 copies/ml for ePlex, and 100 copies/ml for the Xpert Xpress assay (including presumptive positive results) (Table 1).

### Clinical performance.

Clinical testing was performed on 108 retrospective and prospective clinical specimens, and the results were compared to those obtained with the reference standard. Xpert Xpress demonstrated a PPA of 98.3%, followed by ePlex at 91.4% and ID NOW at 87.9%. NPA was also calculated and was 100% for each platform evaluated (Table 2). One sample was invalid on ID NOW and was not included in the calculations for this platform. Further evaluation of distributions of positive results across all three platforms showed that Xpert Xpress detected a total of 57 positive results, followed by ePlex at 53 and ID NOW at 50. ePlex also detected 3 positive results that were not detected by ID NOW, and ID NOW detected 1 positive result that was not detected by ePlex, but all 4 of those positive samples were detected by Xpert Xpress, as well as 4 additional positive samples that were detected only by Xpert Xpress. ePlex and ID NOW did not detect any additional results that were not detected by Xpert Xpress. One specimen that was positive on Panther Fusion was not detected on any of the 3 platforms.

**TABLE 2:** Clinical performance comparison of three sample-to-answer EUA molecular assays for the detection of SARS-CoV-2 (*n* = 108)

Molecular assay	Reference standard^*a*^		Kappa (κ) (±95% CI)^*b*^	PPA (±95% CI)^*b*^	NPA (±95% CI)^*b*^
	Positive	Negative			
Xpert Xpress					
Positive	57	0		98.3	100
Negative	1	50	0.98 (1–0.95)	(0.91–1)	(0.93–1)

ID NOW^*c*^					
Positive	50	0		87.7	100
Negative	7	50	0.87 (0.96–0.78)	(0.76–0.95)	(0.93–1)

ePlex					
Positive	53	0		91.4	100
Negative	5	50	0.91 (0.99–0.83)	(0.81–0.97)	(0.93–1)

a The reference standard was the Hologic Fusion assay. Data represent numbers of patients.

b ±95% CI, upper/lower 95% confidence interval (>0.90, almost perfect; 0.80 to 0.90, strong; 0.60 to 0.79, moderate; 0.40 to 0.59, weak; 0.21 to 0.39, minimal; 0 to 0.20, none).

c ID NOW had one invalid result that was removed from the analysis which was positive by the reference standard and the other two methods.

A total of eight discordant samples were found among the three sample-to-answer platforms evaluated, with ID NOW having the most discordant results (*n* = 7), followed by ePlex (*n* = 5) and Xpert Xpress (*n* = 1). All discordant results were negative results compared to a positive result from the reference method. In evaluating the cycle threshold (*C_T_*) values obtained by the reference method, A-24, which was the only discordant specimen in detection by the Xpert Xpress assay, gave a *C_T_* value of 38.5, which would be considered a positive specimen representing a low viral load. ePlex exhibited negative results with specimens that had *C_T_* values ranging from 33.1 to 38.5, while ID NOW exhibited negative results with specimens that had *C_T_* values that ranged from 32 to 38.5 (Table 3).

**TABLE 3:** Details of discordant samples^*a*^

Sample ID	SARS-CoV-2 sample-to-answer molecular assay results			
	Reference method (*C_T_* value)	Xpert Xpress (E/N2 *C_T_* values)	ID NOW	ePlex
**A-10**	POS (33.1)	POS (32.8/35.8)	POS	**NEG**
**A-12**	POS (33.2)	POS (31.7/34.6)	**NEG**	**NEG**
**A-14**	POS (34)	POS (33.3/35.5)	**NEG**	**NEG**
**A-15**	POS (32.6)	POS (32.2/35.4)	**NEG**	POS
**A-16**	POS (33.2)	POS (33.6/36.4)	**NEG**	POS
**A-24**	POS (38.5)	**NEG (N/A)**	**NEG**	**NEG**
**A-26**	POS (36.2)	POS (36.6/39.5)	**NEG**	**NEG**
**A-103**	POS (32)	POS (31.1/34.3)	**NEG**	POS

a Discordant sample results are highlighted in bold. *C_T_*, cycle threshold; ID, identifier; NEG, negative; POS, positive.

Hands-on time (HoT), run time, and total turnaround time (TAT) per specimen were evaluated. Xpert Xpress HoT is approximately 1 min per specimen, while ID NOW and ePlex both had a HoT of approximately 2 min per specimen. ID NOW had the shortest overall TAT of ∼17 min for one specimen. The Xpert Xpress TAT was ∼46 min for one specimen and the ePlex TAT was ∼1.5 h for one specimen, with the majority of the TAT measured for each assay being assay run time. The ID NOW turnaround times can also differ for positive specimens and can be as low as 5 min, including HoT (Table 4).

**TABLE 4:** Basic performance characteristics of three sample-to-answer EUA molecular SARS-CoV-2 assays evaluated^*a*^

Characteristic	Xpert Xpress SARS-CoV-2	ID NOW COVID-19	ePlex SARS-CoV-2
Manufacturer	Cepheid	Abbott	GenMark
Sample type(s)	NPS, NS, midturbinate swab, nasal wash, nasal aspirate	NPS, NS, TS	NPS
Sample vol required (μl)	300	200	200
Extraction required	Yes (automated)	No	Yes (automated)
Detection platform/system	GeneXpert, Xpress, Infinity	ID NOW	ePlex
Target region of SARS-CoV-2	N2, E	RdRp	N
Analytical sensitivity per claim	250 copies/ml	125 genome equivalents/ml	100,000 RNA transcript copies/ml
Maximum throughput	4 per instrument (4-module configuration)	1 per instrument	6 per tower
Hands-on time (per specimen)	∼1 min	∼2 min	∼2 min
Assay run time	∼45 min	<15 min	∼90 min
User results interpretation	No	No	No
Overall turnaround time (per specimen)	∼46 min	∼17 min	∼1.5 h

a NPS, nasopharyngeal swab; NS, nasal swab; TS, throat swab.

## DISCUSSION

Clinical confirmation of COVID-19 is at the core of our strategy to stop the current spread of infection. It has recently been shown that SARS-CoV-2 has a basic reproduction number (*R*_0_) of 2.2, meaning that an infected person can spread the infection to two additional persons on average (5, 6).

Vulnerable patient populations, such as people with preexisting medical conditions, immunocompromised individuals, and the elderly, especially those living in a nursing home or a long-term-care facility, are especially at risk (11, 12). With this in mind, it is critical that patient results are as accurate as possible and are also available in a rapid fashion to stop the spread of infection in real time.

We evaluated three sample-to-answer platforms currently in use in our health system for the detection of SARS-CoV-2, including Xpert Xpress and ID NOW, which are designed to be used in near-patient testing environments and outside the clinical laboratory environment. LoD determination, correlation of clinical results, and performance comparisons, including HoT and overall TAT, were done for each assay as part of our evaluation. This information is especially critical at the current moment, when accurate and rapid results are at the center of clinical decision-making, both in outpatient clinics and in the hospital. All three of these platforms are designed to produce rapid test results, and each platform is a sample-to-answer system designed to run one patient per test cartridge. This makes the comparison of these platforms especially pertinent as decisions are made for testing in both the inpatient and outpatient environments.

In our comparisons of all three platforms, Xpert Xpress outperformed both ID NOW and ePlex, exhibiting the lowest LoD of all three platforms (100 copies/ml), whereas ID NOW and ePlex had higher LoDs of 20,000 and 1,000 copies/ml, respectively (Table 1). We also observed that in each case, the manufacturer’s stated LoD differed from our findings, with ePlex having a much lower LoD than that stated in their EUA submission (1,000 RNA copies/ml versus the EUA-listed value of 100,000 RNA transcript copies/ml), while ID NOW had a much higher LoD than that stated in their EUA submission (20,000 RNA transcript copies/ml versus the EUA-listed value of 125 genome equivalents/ml). The LoD reported by the manufacturer of Xpert Xpress was the closest to the measured value among those reported by the manufacturers whose products were tested here (100 RNA transcript copies/ml, including presumptive positives, versus the EUA-stated 250 copies/ml). This disparity might have been due to the use of different quantified materials among the manufacturers. Our LoD findings also correlated with the clinical sensitivities, which ranged from a high of 98.3% for Xpert Xpress to a low of 87.9% for ID NOW, with ePlex falling in the middle at 91.4% (Table 2). A closer analysis of positive results showed that whereas the majority of positives were detected by all three platforms, Xpert Xpress detected four results that were missed by both ID NOW and ePlex and also detected additional results singly detected by either ID NOW or ePlex. All three assays had 100% specificity and did not exhibit false-positive results.

When it comes to the HoT and TAT of the three platforms, each platform has specific advantages. Xpert Xpress is the easiest to use and requires the fewest technical interventions, which include loading the sample and the cartridge. ID NOW has the shortest sample-to-answer time at ∼17 min maximum to final result. ePlex has the ability to test higher numbers of patients at once on a random-access 6-bay tower. Both the Xpert Xpress and ePlex platforms can also be expanded by adding modules/bays for more capacity in certain models of instrumentation, while ID NOW is limited to 1 sample testing port per instrument.

Some limitations of this study were that this was a single-center study and the majority of specimens were initially tested on the ePlex system and were then stored frozen. While this was the case, ePlex had performance sensitivity considerably lower than that of the reference standard (Panther Fusion) and of Xpert Xpress and yet had the competitive advantage as the assay that was initially performed on fresh specimens. Considering this workflow limitation, the results of our study in regard to the sensitivity of ePlex are even more telling, since the ePlex results do not contain testing after one freeze-thaw of retrospective specimens, such as was the case for Xpert Xpress and ID NOW (as well as the reference standard).

In addition, while the number of specimens included in the clinical correlation was only 108, these specimens were chosen to span the positivity range of clinical specimens, including those specimens with a low viral load. Also, the percentage of positive specimens in our study actually reflected the overall true positivity rate determined by us (50% to 60% SARS-CoV-2 positive) for this time period.

In summary, we evaluated three sample-to-answer platforms for the detection of SARS-CoV-2 using NP specimens, including two platforms that are designed to be used in the near-patient testing environment, Xpert Xpress and ID NOW. Our results showed that Xpert Xpress performed well and had the lowest LoD and highest sensitivity, while ePlex and ID NOW had lower sensitivities and missed several positive patient specimens. The lack of sensitivity in both ID NOW and ePlex is particularly concerning in the midst of this current pandemic, where identifying new infections is the bedrock of limiting spread. While ID NOW is the most rapid of the three platforms tested, taking ∼17 min from beginning to end to complete testing, it missed 12.3% of the positive patients tested, exhibiting a sensitivity of 87.7% in our study. ePlex also missed 8.6% of positive patients and had a sensitivity of 91.4% and also takes ∼1.5 h to perform. In contrast, Xpert Xpress missed 1.7% of positive patients, showing a sensitivity of 98.3%, and takes ∼46 min to perform. In conclusion, while ID NOW gives the most rapid result, both ID NOW and ePlex (which takes substantially longer to produce results) lack sensitivity compared to Xpert Xpress. These parameters will need to be considered when deciding which testing platform should be implemented for COVID-19 testing.
